# Circulating Antibodies Against Common Cold Coronaviruses Do Not Interfere with Immune Responses to Primary or Booster SARS-CoV-2 mRNA Vaccines

**DOI:** 10.3390/vaccines13050547

**Published:** 2025-05-21

**Authors:** Bindu Adhikari, Eugene M. Oltz, Richard J. Gumina, Maryssa K. Kick, Linda J. Saif, Anastasia N. Vlasova

**Affiliations:** 1 Department of Veterinary Preventive Medicine, College of Veterinary Medicine, The Ohio State University, Wooster, OH 44691, USA; adhikari.120@osu.edu (B.A.); saif.2@osu.edu (L.J.S.); 2 Center for Food Animal Health, Department of Animal Sciences, OARDC, College of Food, Agricultural and Environmental Sciences, The Ohio State University, Wooster, OH 44691, USA; kick.28@osu.edu; 3 Department of Microbial Infection and Immunity, The Ohio State University, Columbus, OH 43210, USA; eugene.oltz@osumc.edu; 4 Department of Internal Medicine, Division of Cardiovascular Medicine, The Ohio State University, Columbus, OH 43210, USA; richard.gumina@osumc.edu

**Keywords:** SARS-CoV-2, common cold coronavirus, antibodies, IgG4, COVID-19 mRNA vaccines

## Abstract

Background: Pre-existing cross-reactive antibodies (Abs) against common cold coronaviruses (CCCoVs) have been hypothesized to influence the immune responses to SARS-CoV-2 vaccine-induced Ab responses. Methods: Serum samples from healthy healthcare workers (HCWs, n = 64) receiving mRNA vaccines were collected at seven time points: pre-COVID-19-vaccination (Pre), post-first dose (Vax1), post-second dose (Vax2), and 6-, 9-, 12-, and 15-months post-Vax2. Booster vaccine doses (n = 23) were received 1–80 days prior to the 9 m sample collection time point. We used peptide-based enzyme-linked immunosorbent assays (ELISAs) to measure SARS-CoV-2/CCCoV-specific IgG/IgA/IgM and SARS-CoV-2 IgG4 (associated with immune tolerance) Ab levels in the HCW serum samples. Additionally, we measured Epstein–Barr/influenza A (unrelated pathogens) virus-specific IgG Ab levels. Results: We observed that vaccination significantly increased SARS-CoV-2 IgG Ab levels at the Vax1 (*p* ≤ 0.0001) and Vax2 (*p* ≤ 0.0001) time points compared to Pre-Vax. These Ab levels declined at 6 months post-vaccination but increased again following the booster vaccine dose around the 9-month post-Vax2 time point in a cohort (n = 23) of the HCWs. However, this increase was modest compared to those induced by the primary vaccine series. Interestingly, a moderate but continuous increase in SARS-CoV-2 S IgG4 Ab levels was observed throughout this study, becoming statistically significant by the 15-month time point (*p* = 0.03). Further, a significant increase in CCCoV IgG (but not IgA/IgM) Ab levels was observed at the Vax1 time point, suggestive of cross-reactive or non-specific immune responses. Finally, we observed no negative correlation between the levels of pre-existing CCCoV-specific Abs and the vaccine-induced Ab response (Vax1/Vax2). Conclusions: Pre-existing CCCoV Abs do not interfere with the development of vaccine-induced immunity. However, vaccine-associated Abs wane over time, which may be associated with the increasing IgG4 Ab response.

## 1. Introduction

The COVID-19 pandemic, caused by severe acute respiratory syndrome-2 (SARS-CoV-2), has resulted in significant morbidity and mortality as well as economic losses globally [1,2]. All human coronaviruses (HCoVs) belong to the *Alphacoronavirus* and *Betacoronavirus* genera [3]. There are seven known HCoVs: 229E, OC43, NL63, SARS-CoV-1, HKU1, MERS-CoV, and SARS-CoV-2 [4,5,6,7,8,9,10]. These HCoVs mainly target the respiratory system and differ in the severity of disease they cause [11]. SARS-CoV-1, MERS-CoV, and SARS-CoV-2 are associated with severe diseases, including COVID-19 and acute respiratory distress syndrome [3,12]. In contrast, 229E, NL63, OC43, and HKU1 typically only cause mild disease, responsible for 15–30% of cases of the common cold [13]. Common cold-causing coronaviruses (CCCoVs) 229E and NL63 belong to the *Alphacoronavirus* genus, whereas HKU1 and OC43 belong to the *Betacoronavirus* genus [12].

Multiple serological studies have been conducted to evaluate the characteristics of Ab responses to SARS-CoV-2 and the influence of the pre-existing cross-reactive immune response against CCCoVs [14,15,16,17,18,19,20,21,22,23,24,25,26,27,28]. However, the potential for positive and negative immunological interactions between CCCoV and SARS-CoV-2 Abs [13,14,15,16,17,18,19,20,21,22,23,24,25,26,27,28,29,30,31], as well as the impact of pre-existing immunity to CCCoVs on Ab responses induced by SARS-CoV-2 natural infection or vaccination, remain critical yet unresolved issues. Therefore, we hypothesize that pre-existing cross-reactive Abs against endemic CCCoVs may interfere with SARS-CoV-2 vaccine-induced Ab responses; alternatively, pre-existing cross-reactive Abs might boost SARS-CoV-2-specific Ab responses.

To test our hypothesis, we analyzed the association between different Ab isotypes targeting SARS-CoV-2 and CCCoVs nucleocapsid (N) and spike (S) proteins in a cohort of healthy healthcare workers (HCWs). Our study, conducted among HCWs vaccinated with COVID-19 mRNA vaccines, aimed to elucidate the influence of pre-existing CCCoV immunity on SARS-CoV-2 vaccine-induced immunity. By assessing Ab responses and their impact over time, our project generated valuable insights regarding the interplay between CCCoVs and SARS-CoV-2-specific Ab responses. In addition to the main objectives, this study also examined the levels of Abs against unrelated antigens/viruses and SARS-CoV-2 S-specific IgG4 Ab levels to examine the specificity and the mechanisms regulating the waning of the vaccine-induced Ab response.

## 2. Materials and Methods

**Figure 1:**
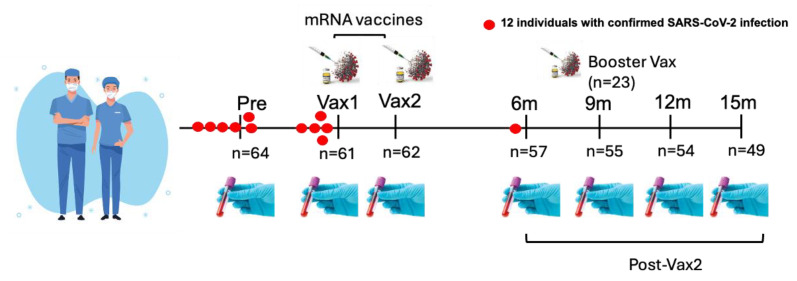
Study design illustrating the longitudinal analysis of antibody (Ab) responses in healthcare workers (HCWs) following mRNA COVID-19 vaccination. Serum samples were collected at seven time points: pre-vaccination (Pre), after the first dose of the vaccine (Vax1), after the second dose of the vaccine (Vax2), and at 6 months (6 m), 9 months (9 m), 12 months (12 m), and 15 months (15 m) post-vaccination. Participants received either the mRNA-1273 or BNT162b2 mRNA vaccine. The sample size for each time point is indicated. The red dots represent individuals who were confirmed to have SARS-CoV-2 infection at specific time points. Booster vaccine doses (n = 23) were received 1–80 days prior to the 9 m sample collection time point.

Serum samples were obtained from sixty-four healthy HCWs. These samples were collected at seven different time points: pre-vaccination (Pre, n = 64), post-vaccination [after the first (Vax1, n = 61) and second (Vax2, n = 62) doses], and at 6- (6 m, n = 57), 9- (9 m, n = 55), 12- (12 m, n = 54), and 15-months (15 m, n = 49) post-vaccination (Table 1 and Figure 1). The sample collection period spanned from April 2020 to May 2022. The Vax1 samples were collected 13–33 days (median = 21; interquartile range (IQR) = 19 to 23) following the first vaccine dose, while Vax2 sampling took place 20–31 days (median = 26; IQR = 22 to 28) following the second vaccination. The HCWs received either mRNA-1273 (Moderna Inc., Cambridge, MA, USA) (n = 29) or BNT162b2 (Pfizer Inc., New York, NY, USA, partnered with BioNTech) (n = 35) vaccines. A total of 23 individuals received a booster vaccine dose, including BNT162b2 (n = 17) and mRNA-1273 (n = 6), around 1–80 days (median = 18; IQR = 7 to 29) prior to the 9 m time point. Additionally, 2 individuals received a second booster dose, both of which were BNT162b2; the time frame for the second booster dose is not available. As individual vaccination records were not available, we assume the standard 2.5 mL (50 μg) dose recommended at that time was used, unless otherwise noted.

The cohort consisted of ages 25 to 61 (median = 36; IQR = 31 to 45) and included 33 males and 31 females. Out of 12 individuals with SARS-CoV-2 infections confirmed by reverse transcription polymerase chain reaction (RT-PCR) during the study period, 10 were infected between 1 and 9 months prior to the 1st vaccine dose. One individual received a vaccine and was infected and tested positive for SARS-CoV-2 within 12 days post-Vax1 (but prior to Vax 1 sampling), while another individual had a breakthrough infection (approximately 6 months post-Vax2). Apart from that, the data regarding the CCCoV/SARS-CoV-2 infection status of these individuals throughout the study period were not available. No additional demographic or clinical data were collected for these HCWs. This study was approved by The Ohio State University’s institutional review board committee (approval number 2020H0198).

Enzyme-linked immunosorbent assays (ELISAs) were employed to measure Ab levels specific to the S and N proteins of alphaCoVs (NL63 and 229E) and betaCoVs (OC43, HKU1, and SARS-CoV-2). As described previously, the 10 CoV-specific peptides used as ELISA antigens were designed to target unique regions of the S and N proteins [32] of SARS-CoV-2 and each CCCoV. The CCCoV S peptides were located within the receptor-binding domain (RBD), while the SARS-CoV-2 S peptide was derived from the S2 region.

Additionally, two more peptides (ALPHA and BETA) were designed to target cross-reactive regions of the N protein of alpha and beta CoVs, respectively [32]. The peptides used in this study, targeting highly antigenic epitopes of the nucleoprotein (N) and spike (S) proteins of SARS-CoV-2 and common cold coronaviruses (CCCoVs), are comprehensively described in our prior publication [32]. The ELISA protocols for N and S protein peptide-based assays were developed and validated as detailed in our previous study [32]. Plasma samples from the cohort were assayed for the presence of Abs against CCCoVs and SARS-CoV-2 using these previously established ELISA protocols [32]. Nunc MaxiSorp 96-well plates were coated with 800 ng/well of each peptide in 1× phosphate-buffered saline (PBS, pH 7.4, Millipore, Burlington, MA, USA). Plasma dilutions with 5% non-fat dry milk (NFDM) in PBS with 0.1% Tween (PBS-T; Polysorbate 20, VWR, Solon, OH, USA) were prepared for CCCoVs: a 1:100 dilution was used, while for SARS-CoV-2, serial 4-fold dilutions starting at 1:100 were used. Horseradish peroxidase (HRP)-conjugated goat anti-human Fc cross-absorbed Ab was added at the manufacturer’s recommended dilutions (IgG 1:2000, IgM 1:1000, IgA 1:1000) in 5% NFDM in PBS-T. The development of the plates followed previously described methods, and optical density (OD) values were measured at 650 nm using SoftMax Pro 7.1 software (Molecular Devices, LLC, San Jose, CA, USA). For SARS-CoV-2-specific ELISAs, cutoff values were established as previously described [32]. The cutoff values were established by calculating the mean OD of negative controls, adding three times the standard deviation to account for variability, and establishing the threshold for positive detection. This method ensured that the cutoffs were stringent and minimized false-positive results.

We also examined the concentrations of total IgG Abs in the samples collected at the Pre and Vax1 time points using an ELISA Flex kit (MabTech, Inc., Cincinnati, OH, USA) for the quantitative determination of native human IgG in the plasma sample (Pre and Vax1). The protocol was followed as per the manufacturer’s guidelines. First, 100 μL of capture mAbs MT145, diluted to 2 μg/mL in PBS (pH 7.4), was added to Nunc MaxiSorp plates, which were then incubated overnight at 4 °C. The next day, the plates were emptied, and 200 μL/well of PBS-T and 0.1% BSA was added to each well as a blocking reagent, followed by 1 h incubation at room temperature. The plates were then washed five times with 0.05% PBS-T (300 μL/well). Next, 100 μL/well of the plasma samples or a standard (a calibrated human IgG) diluted in 0.05% PBS-T was added and incubated for 2 h at room temperature. The plates were washed again, as described above. Then, 100 μL/well of detection mAB MT78-biotin, diluted to 0.25 μg/mL in 0.05% PBS-T, was added and incubated for 1 h at room temperature, followed by another wash. Subsequently, 100 μL/well of Strepavidin-HRP, diluted 1:1000 in 0.05% PBS-T, was added and incubated for 1 h at room temperature. After the final wash, 100 μL/well of TMB substrate was added and incubated for 15 min. The reaction was stopped by adding 100 μL/well of 0.2 M H_2_SO_4_. The OD values were measured, and we compared the total IgG Ab concentrations of the samples using SoftMax Pro 7.1 software (Molecular Devices, LLC, San Jose, CA, USA) at 450 nm within 15 min.

Additionally, we also examined the levels of IgG Abs targeting unrelated antigens/viruses [Epstein–Barr (EBV) and influenza A (IAV, H1N1 and H3N2) viruses] in the samples collected at the Pre and Vax1 time points. The ELISAs were conducted as previously described [32]. Briefly, the wells of 96-well Nunc MaxiSorp plates were coated with 800 ng/well of target protein in 1× PBS (pH 7.4) overnight at 4 °C. Target proteins included EBV glycoprotein 350 (gp350, Sinobiological, Wayne, PA, USA) and influenza A H1N1 (Brisbane/2018) and H3N2 (Cambodia/2020) hemagglutinin proteins (HA, Sinobiological). The plates were then rinsed and blocked, and plasma dilutions were prepared at 1:100. These dilutions were prepared with 5% NFDM in PBS-T, and 50 μL of the plasma was added in duplicate wells and incubated at 37 °C for 45 min. The plates were then washed five times with 0.05% PBS-T. Next, 50 µL HRP-conjugated goat anti-human Fc cross-absorbed Ab was added at the manufacturer’s recommended dilutions (IgG 1:2000) in 5% NFDM in PBS-T. After a 45 min incubation at 37 °C, the plates were washed five times with 0.05% PBS-T. The development of the plates followed previously described methods, and OD values were measured at 650 nm using SoftMax Pro 7.1 software (Molecular Devices, LLC, San Jose, CA, USA).

Additionally, we measured the levels of SARS-CoV-2 S IgG4 Abs. Similar to the protocol described above, 96-well Nunc MaxiSorp plates were coated with 800 ng/well of SARS-CoV-2 S peptide in 1× PBS (pH 7.4) and incubated overnight at 4 °C. The plates were then rinsed and blocked, and plasma serial 4-fold dilutions were prepared starting at 1:100. These dilutions were prepared with 5% NFDM in PBS-T, and 50 μL of the plasma was added in duplicate wells and incubated at 37 °C for 45 min. The plates were then washed five times with 0.05% PBS-T. Next, 50 µL of HRP-conjugated mouse anti-human IgG4 Fc cross-absorbed Ab from the Thermo Fisher Scientific catalog (**#A-10654**) was added at the manufacturer’s recommended dilution (IgG4 1:1000) in 5% NFDM in PBS-T. After a 45 min incubation at 37 °C, the plates were washed five times with 0.05% PBS-T. The development of the plates followed previously described methods, and OD values were measured at 650 nm using SoftMax Pro 7.1 software (Molecular Devices, LLC., San Jose, CA, USA). Cutoff values were established as previously described [32].

We conducted statistical analyses using PRISM 10.4.1 (GraphPad Software, Inc., San Diego, CA, USA). To compare unpaired values, Mann–Whitney and Kruskal–Wallis post hoc tests adjusted with Dunn’s test for multiple comparisons were performed, ensuring accurate control of Type I error rates across pairwise comparisons. Pearson’s rank correlation was used for correlation studies. *p*-values < 0.05 were set for significance.

## 3. Results

**Figure 2:**
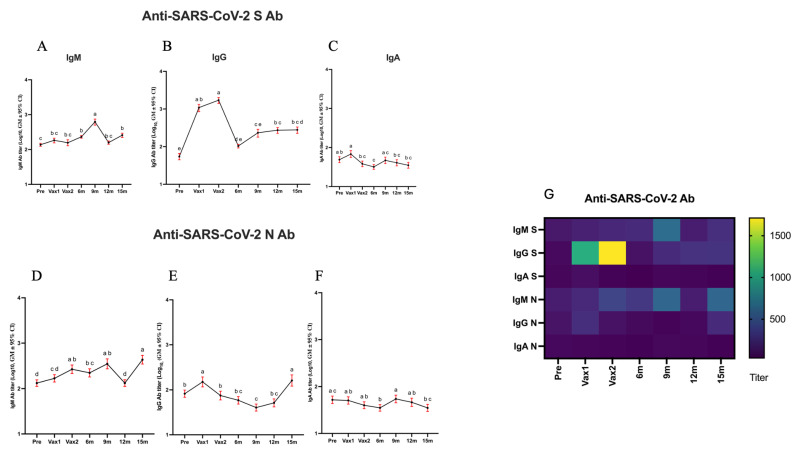
The line graphs (**A**–**F**) represent the temporal dynamics of antibody (Ab) responses (IgM, IgG, and IgA) against two SARS-CoV-2-derived peptides: spike (S) and nucleocapsid (N). SARS-CoV-2 S (**A**–**C**) and N (**D**–**F**) protein-specific IgM/IgG and IgA Ab geometric mean titers measured using S and N protein peptide ELISAs. The *X*-axis denotes the seven time points: pre-vaccination (Pre), first vaccine dose (Vax1), second vaccine dose (Vax2), 6 months post-vaccination (6 m), 9 months post-vaccination (9 m), 12 months post-vaccination (12 m), and 15 months post-vaccination (15). The *Y*-axis indicates the SARS-CoV-2 Ab titer. Each line allows for a direct comparison of the Ab response across time points. Different letters indicate significant differences. Different time points that share the same letter are not significantly different from each other, while the time points with different letters indicate significant differences (*p* < 0.05). Log_10_-transformed antibody titers are presented as geometric mean ± 95% confidence interval (CI). The heatmap (**G**) complements the line graph by visually summarizing the Ab response (IgM, IgG, and IgA) against S and N peptides across the same seven time points. The *X*-axis represents the time points, while the *Y*-axis lists the Ab–peptide combinations. The color gradient illustrates the Ab titer, with darker purple indicating lower levels and brighter yellow representing higher titers. Cutoff values were calculated as the mean of negative controls + 3 standard deviations.

To understand the dynamics of SARS-CoV-2 vaccine-induced immune responses in healthy individuals, we compared the levels of serum IgG/IgA/IgM Abs against the SARS-CoV-2 S and N proteins in a cohort of healthy HCWs before and after COVID-19 vaccination. We observed that vaccination significantly increased SARS-CoV-2 S IgG Ab levels across multiple time points (Figure 2B). Specifically, the post-vaccination (Vax1 and Vax2) SARS-CoV-2 S protein-specific IgG Ab levels were significantly increased compared with those pre-vaccination (Pre) (Figure 2B,G). However, the Ab titers dropped significantly by the 6 m time point while still remaining higher than at the Pre time point (Figure 2B). Additionally, we observed that the SARS-CoV-2 S-specific IgG Ab titers increased significantly again around the 9 m time point (following a booster vaccine dose received by some HCWs; Figure S1A), but this increase was modest compared to those induced by the primary vaccine doses. These findings underscore that while both vaccine doses as well as booster vaccination induced significant increases in SARS-CoV-2 S IgG Ab levels (Figure 2B and Figure S1A), they waned over time, and repeated booster immunizations may have more limited immunogenicity than the primary vaccine series. Alternatively, this could be associated with the lower immunogenicity/increased immunoevasive potential of the Omicron variants circulating during the second year of this study. It is also possible that after repeated vaccinations and infections, the immune system more efficiently prevents new COVID-19 infections, thus reducing antigenic stimulation and the associated immune response. Additionally, the booster vaccination was associated with significantly higher IgG Ab titers in the short term (at the 9 m time point), and these titers were largely maintained through the 12 m and 15 m time points (Figure S1). At 9 m, individuals who received a booster vaccination and were infected (boosted + infected) had the highest Ab titers compared to the other groups, with a statistically significant difference observed compared to the infected + non-boosted group (Figure S1B). However, at 12 m, the pattern was reversed, with the infected + non-boosted group showing higher Ab titers than the boosted + infected group (Figure S1B). This highlights the dynamic nature of immune responses following booster vaccination and natural infection. At 9 m, the enhanced Ab titers in the boosted + infected group indicate the synergistic effect of booster vaccination and natural infection in amplifying the immune response. This finding is consistent with previous studies showing that hybrid immunity generates robust Ab levels in the short term [33,34]. However, at the 12 m and 15 m time points, the observed decline in the Ab titers in the boosted + infected group suggests a more rapid waning of immunity compared to the infected + non-boosted group. This could reflect a natural plateau or exhaustion of the immune system following repeated antigenic stimulation. In contrast, individuals in the infected + non-boosted group maintained or slightly increased their Ab levels, potentially due to the sustained effects of natural infection-induced immunity.

Surprisingly, there was a significant increase in SARS-CoV-2 N protein-specific IgG Ab levels following the first vaccination, regardless of prior infection, indicative of cross-reactive or non-specific Ab response (Figure 2E). Considering the concurrent increases in the levels of CCCoV anti-S-specific and anti-N-specific Abs, this could be associated with undiagnosed CCCoV infections. It is important to note that the magnitude of this increase and the overall titers of the SARS-CoV-2 N-specific Abs were much lower than those directed against the SARS-CoV-2 S protein (Figure 2E). Additionally, in contrast to the SARS-CoV-2 S-specific IgG Ab levels, there was no further increase in the N-specific IgG Ab levels following the second vaccine dose, consistent with the use of S-based mRNA vaccines. However, they increased at the 15 m time point, as did IgM S and N Abs, which could be suggestive of recent or ongoing SARS-CoV-2 or CCCoV infections that remained undocumented or asymptomatic (Figure 2D,E). We also compared the levels of serum IgG Abs against the SARS-CoV-2 S protein based on mRNA vaccine type (mRNA-1273 vs. BNT162b2) and observed no significant differences associated with these two vaccines at any time point (Figure S2).

While the SARS-CoV-2 IgA Ab levels were much lower compared to IgG (Figure 2C,F), there was an increase in the former between the Pre and Vax1 time points (Figure 2C). This was followed by significant decreases at the Vax2, 6 m, 12 m, and 15 m time points. There was no increase in the SARS-CoV-2 IgA N-specific Ab levels following the first vaccine dose, or at the other Vax2, 6 m, and 15 m time points (Figure 2F). Finally, there were modest increases in the SARS-CoV-2 anti-S and anti-N IgM Ab titers/levels throughout this study; however, the IgM Ab titers dropped at the 12 m time point (Figure 2A,D). Significant increases in the SARS-CoV-2 N-specific IgM Ab titers were also evident at the Vax2, 9 m, and 15 m time points compared to the Pre as well as the Vax1 time points, which could be suggestive of recent or ongoing SARS-CoV-2 or CCCoV infections that remained asymptomatic. Overall, these results demonstrate that the COVID-19 mRNA vaccines induced robust SARS-CoV-2 S protein-specific IgG but lower IgA and IgM Ab responses.

**Figure 3:**
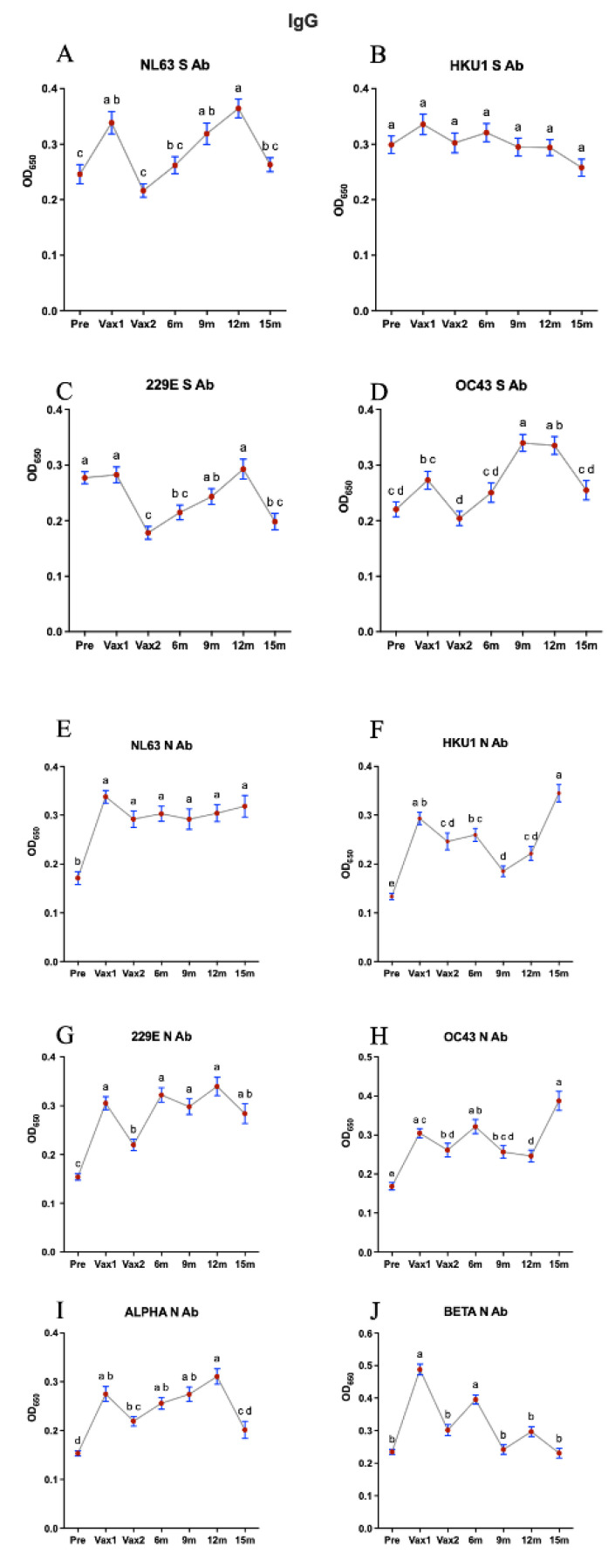
This figure illustrates the optical density (OD) values representing IgG antibody (Ab) levels against conserved and specific antigens of seasonal common cold coronaviruses (CCCoVs) across seven time points: pre-vaccination (Pre), first vaccine dose (Vax1), second vaccine dose (Vax2), 6 months post-vaccination (6 m), 9 months post-vaccination (9 m), 12 months post-vaccination (12 m), and 15 months post-vaccination (15 m). Serum samples were diluted 1:100 for the detection of the IgG antibody against CCCoVs. The left column displays IgG Ab levels against alpha CCCoVs (**A**,**C**,**E**,**G**), while the right column represents beta CCCoVs (**B**,**D**,**F**,**H**). Alpha CCCoV-specific IgG Ab levels and beta CCCoV-specific IgG Ab levels were measured using S protein (RBD) and N protein peptide ELISAs. The graphs are subdivided into anti-S Ab levels (top two rows) and anti-N Ab levels (bottom two rows) for each virus. Additionally, the bottom-most row shows IgG Ab levels against conserved N peptides for alpha CCCoVs (**I**) and beta CCCoVs (**J**). The red dots indicate individual data points, with lines connecting mean OD values across time points; different letters indicate statistically significant differences. Different time points that share the same letter are not significantly different from each other, while the time points with different letters indicate significant differences (*p* < 0.05). Data are presented as mean ± SEM.

**Figure 4:**
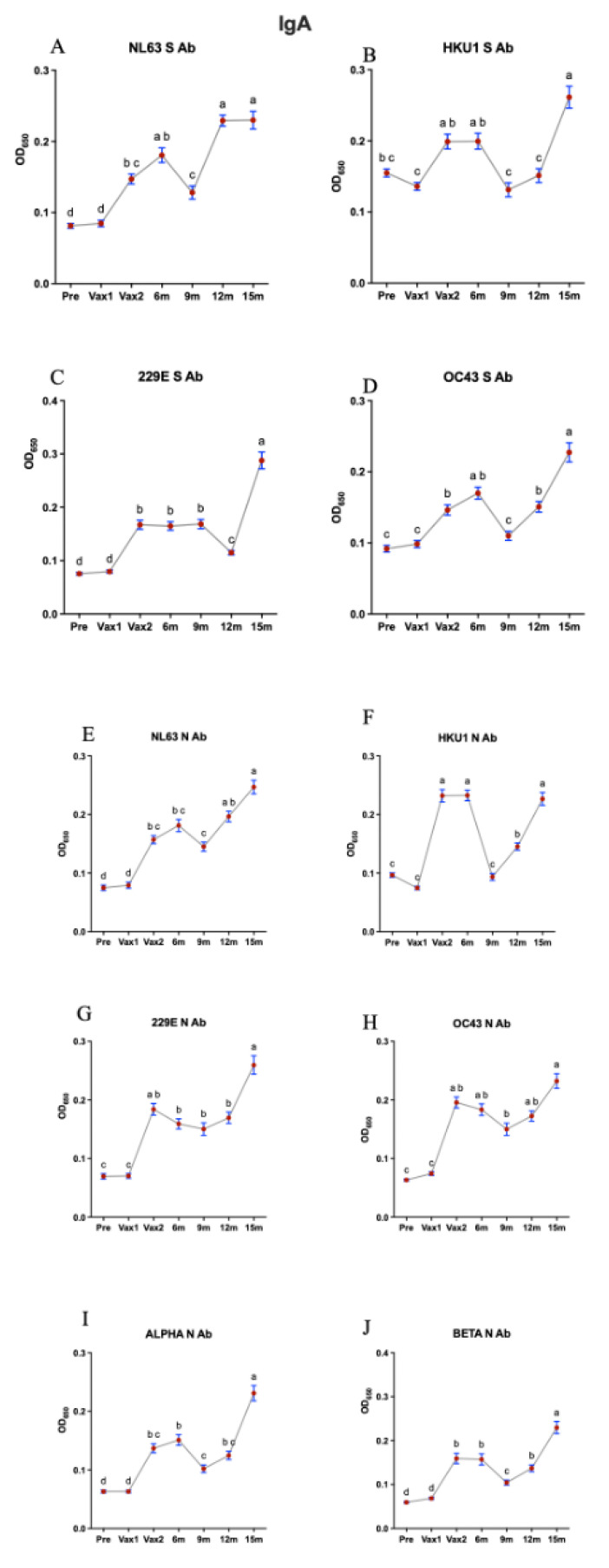
This figure illustrates the optical density (OD) values representing IgA antibody (Ab) levels against conserved and specific antigens of seasonal common cold coronaviruses (CCCoVs) across seven time points: pre-vaccination (Pre), first vaccine dose (Vax1), second vaccine dose (Vax2), 6 months post-vaccination (6 m), 9 months post-vaccination (9 m), 12 months post-vaccination (12 m), and 15 months post-vaccination (15 m). Serum samples were diluted 1:100 for the detection of the IgG antibody against CCCoVs. The left column displays IgA Ab levels against alpha CCCoVs (**A**,**C**,**E**,**G**), while the right column represents beta CCCoVs (**B**,**D**,**F**,**H**). Alpha CCCoV-specific IgA Ab levels and beta CCCoV-specific IgA Ab levels were measured using S protein (RBD) and N protein peptide ELISAs. The graphs are subdivided into anti-S Ab levels (top two rows) and anti-N Ab levels (bottom two rows) for each virus. Additionally, the bottom-most row shows IgA Ab levels against conserved N peptides for alpha CCCoVs (**I**) and beta CCCoVs (**J**). The red dots indicate individual data points, with lines connecting mean OD values across time points; different letters indicate statistically significant differences. Different time points that share the same letter are not significantly different from each other, while the time points with different letters indicate significant differences (*p* < 0.05). Data are presented as mean ± SEM.

**Figure 5:**
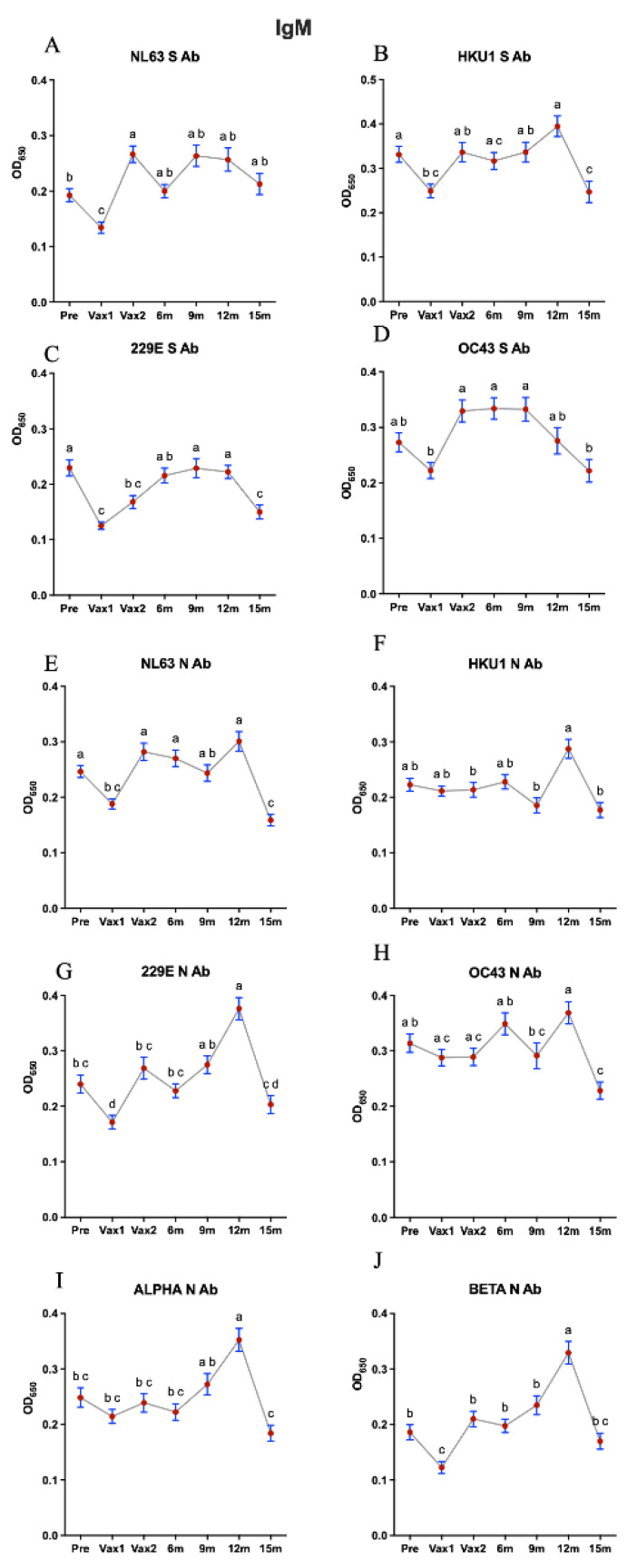
This figure illustrates the optical density (OD) values representing IgM antibody (Ab) levels against conserved and specific antigens of seasonal common cold coronaviruses (CCCoVs) across seven time points: pre-vaccination (Pre), first vaccine dose (Vax1), second vaccine dose (Vax2), 6 months post-vaccination (6 m), 9 months post-vaccination (9 m), 12 months post-vaccination (12 m), and 15 months post-vaccination (15 m). Serum samples were diluted 1:100 for the detection of IgG antibody against CCCoVs. The left column displays IgM Ab levels against alpha CCCoVs (**A**,**C**,**E**,**G**), while the right column represents beta CCCoVs (**B**,**D**,**F**,**H**). Alpha CCCoV-specific IgM Ab levels and beta CCCoV-specific IgM Ab levels were measured using S protein (RBD) and N protein peptide ELISAs. The graphs are subdivided into anti-S Ab levels (top two rows) and anti-N Ab levels (bottom two rows) for each virus. Additionally, the bottom-most row shows IgM Ab levels against conserved N peptides for alpha CCCoVs (**I**) and beta CCCoVs (**J**). The red dots indicate individual data points, with lines connecting mean OD values across time points; different letters indicate statistically significant differences. Different time points that share the same letter are not significantly different from each other, while the time points with different letters indicate significant differences (*p* < 0.05). Data are presented as mean ± SEM.

Next, we measured and compared the levels of serum IgG/IgA/IgM Abs against CCCoV S and N proteins. Similar to our findings on the SARS-CoV-2 N-specific IgG Ab responses, we observed that the first vaccine dose induced a significant increase (although low-level and short-lived) in the levels of all CCCoV N-specific IgG Abs, suggestive of the N-protein-mediated cross-reactive or possibly induced by the mRNA vaccine lipid-nanoparticle-mediated inflammatory response [35,36] (Figure 3). Additionally, the levels of NL63 S-specific IgG Abs were increased significantly following the first vaccination and for NL63 and OC43 at 9 m and 12 m compared with pre-Vax or Vax 1, respectively. There was also a significant decrease in the anti-N and anti-S CCCoV IgG Ab levels following the second vaccine dose (except for HKU1 S and NL63 N Abs), which was likely associated with the Ab affinity maturation (Figure 3). This finding is significant as it was generated using previously developed assays with confirmed high specificity, ensuring the accuracy of our results [32]. Consistent with the relatively low concentration in serum and high affinity of IgA Abs, we observed that the levels of CCCoV-specific IgA Abs were 2–2.5 times lower than those of CCCoV-specific IgG Abs (Figure 3 and Figure 4). The CCCoV IgA Ab levels increased significantly by the Vax2 time point, compared with the Pre or Vax1 levels, then waned to the lowest levels at 9 m (12 m for 229E S Ab), and another significant increase in their levels was noted at the 15 m time point (Figure 4). As explained later, these IgA Ab fluctuations could be associated with CCCoV infections (not monitored in this study) rather than with the SARS-CoV-2 infections reported for some individuals. Finally, we did not observe distinct or pronounced trends for the CCCoV-specific IgM Ab levels with the exception of significant decreases at the Vax1 (except for HKU1 and OC43 N Ab) and 15 m time points (Figure 5). IgM S and N Abs increased for different CCCoVs at 6, 9, or 12 m.

**Figure 6:**
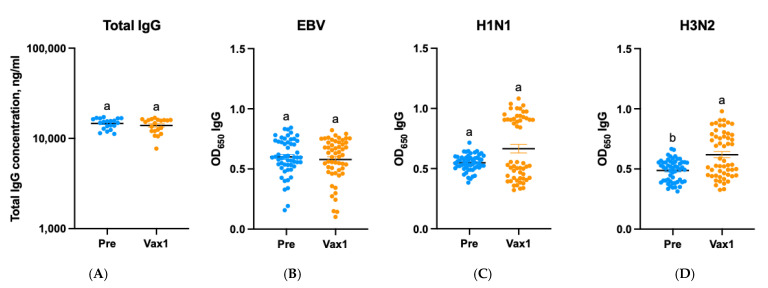
The concentrations of total IgG Abs (**A**) and the levels of IgG Abs targeting unrelated antigens/viruses [Epstein–Barr (EBV) and influenza A (H1N1 and H3N2) viruses] (**B**–**D**); different letters indicate statistically significant differences.

To understand the observed unexpected increases in the N protein-specific IgG Abs at the Vax1 time point, we examined the concentrations of total IgG Abs and the levels of IgG Abs targeting unrelated antigens/viruses [EBV gp350 and IAV (H1N1 and H3N2) HA] in the samples collected at the Pre and Vax1 time points. These Abs are commonly found at variable levels in most adults. Our results demonstrated that while there were no increases in the concentrations/levels of total or EBV-specific IgG Abs, the levels of IAV-specific IgG Abs were increased (significantly for H3N2) (Figure 6). Thus, these findings suggest that it is not a generalized immune activation following the first COVID-19 vaccine dose, but rather a low-level immunological cross-reactivity that goes beyond other CoVs and the S protein. Additionally, we cannot rule out that some of the study participants could have received IAV vaccines.

**Figure 7:**
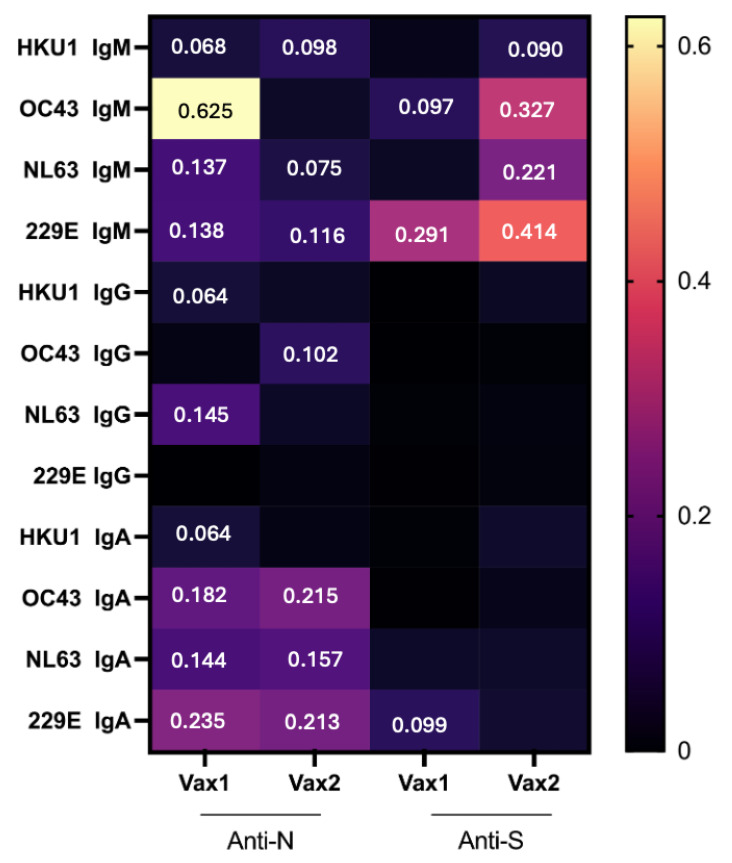
Correlation analysis between SARS-CoV-2 and CCCoVs. Significant (*p* ≤ 0.05) Pearson’s correlation coefficients are presented in the heatmap. The analysis includes data from two vaccination groups, Vax1 and Vax2, and measures of antibody levels targeting anti-S and anti-N SARS-CoV-2 proteins. Each cell represents the correlation coefficient between the respective parameters, with higher coefficients (closer to 1) indicating stronger positive correlations. The color gradient reflects the strength of correlation, ranging from weak (dark purple) to strong (bright yellow or orange).

To understand whether pre-existing CCCoV Ab responses can interfere with the generation of SARS-CoV-2 vaccine-induced Abs, we examined whether there was a correlation between CCCoV and SARS-CoV-2 IgM, IgA, and IgG Ab levels. Our results clearly demonstrated that there was no negative correlation between the vaccine-induced Ab response to SARS-CoV-2 (post-Vax1/Vax2) and the levels of pre-existing CCCoV-specific Abs (Figure 7). Instead, there was a weak-to-moderate positive correlation between some CCCoV and SARS-CoV-2 Ab levels. Of interest, the strongest correlation (e.g., highest Pearson’s correlation coefficient values) was observed between the OC43 and SARS-CoV-2 IgM N Abs at Vax1, followed by the OC43/229E and SARS-CoV-2 S-specific IgM Ab levels at the Vax2 time point (Figure 7). This was followed by correlations between the CCCoV and SARS-CoV-2 N protein-specific IgA and IgM levels, while the IgG N or S protein-specific Ab levels showed the least or no correlation. Because the SARS-CoV-2 vaccines specifically induced IgG Abs against the SARS-CoV-2 S protein, expectedly, these Ab levels did not seem to correlate with low levels of CCCoV Abs. In contrast, the variable levels of SARS-CoV-2/CCCoV anti-N/S IgM and anti-N IgA were likely associated with natural infections or representative of the overall immune function and correlated with one another.

**Figure 8:**
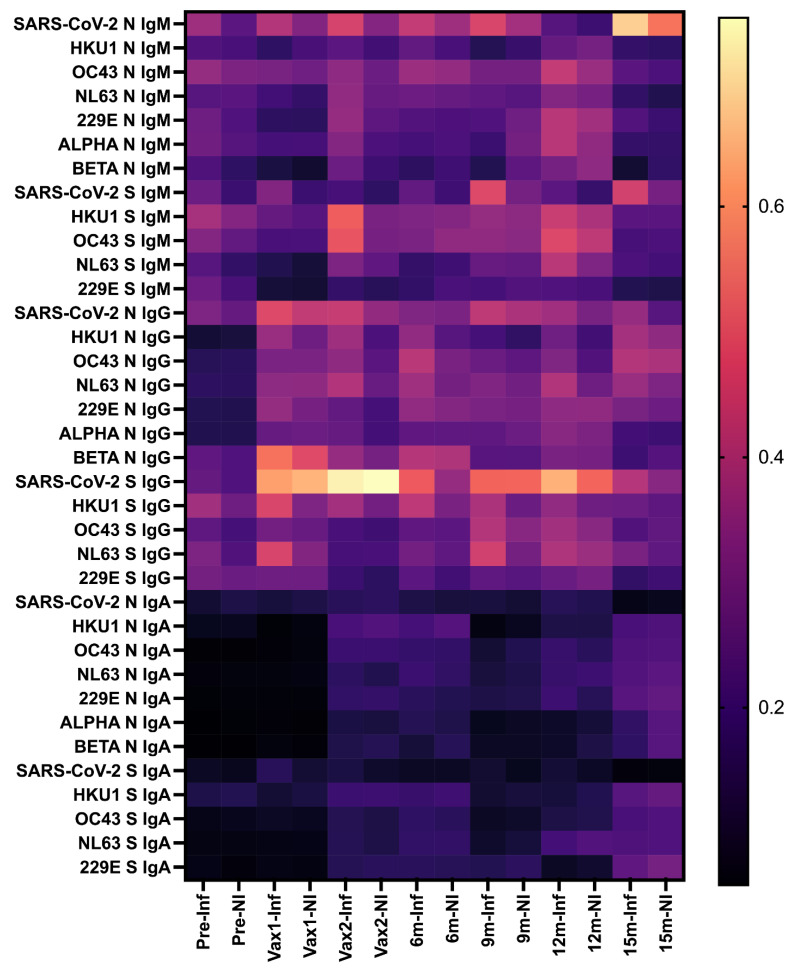
Heatmap showing SARS-CoV-2/CCCoV-specific IgM/IgG/IgA Ab levels measured using S protein and N protein peptide ELISAs in infected (Inf) and non-infected (NI) individuals. The color gradient reflects the strength of correlation, ranging from weak (dark purple) to strong (bright yellow or orange).

Our next goal was to understand whether the confirmed SARS-CoV-2 infections (reported for 12 individuals, as described in the Materials and Methods) had an impact on the SARS-CoV-2 and CCCoV Ab levels prior to and after the vaccination. Our results demonstrated that infected (Inf) individuals had increased levels of SARS-CoV-2-specific IgG and IgM Abs, as well as cross-reactive IgG and IgM Abs targeting some CCCoVs compared to non-infected (NI) individuals (Figure 8).

Of interest, mRNA vaccination was associated with increased levels of cross-reactive IgM Abs against HKU1 and OC43 S proteins (at Vax2 time point) and IgG Abs against HKU1 and NL63 S proteins (at Vax1 time point) in individuals with confirmed SARS-CoV-2 infection. Another unexpected trend was the low levels but generally steady increase in the CCCoV IgA Ab levels that contrasted with the trend observed for SARS-CoV-2 IgA Abs. This could be due to ongoing CCCoV infections that were not monitored for this cohort. In contrast, the increase in SARS-CoV-2 N and S protein-specific IgM at the end of this study could be explained by mild/asymptomatic SARS-CoV-2 infections that occurred but were not confirmed in some patients. Overall, while confirmed SARS-CoV-2 infections were clearly associated with increased levels of SARS-CoV-2/CCCoV N- and S-specific IgG and IgM Abs, SARS-CoV-2 vaccination alone also seemed to boost these Ab levels, but mild/asymptomatic SARS-CoV-2/CCCoV infections (not diagnosed) could also contribute to these results.

**Figure 9:**
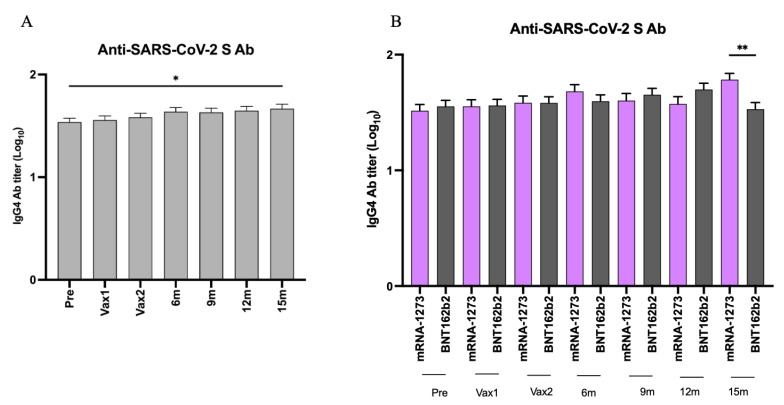
SARS-CoV-2 S protein-specific IgG4 Ab titers across different time points (**A**). SARS-CoV-2 S protein-specific IgG4 Ab titers in individuals who received mRNA-1273 vs. BNT162b2 mRNA vaccines across different time points (**B**). The symbol (*) denotes a *p*-value < 0.05, while (**) represents *p*-value < 0.01, indicating statistically significant differences. Log_10_-transformed antibody titers are presented as geometric mean ± 95% confidence interval (CI).

Finally, to understand the reason behind the waning of the vaccine-induced SARS-CoV-2 Abs and the modest Ab response associated with the booster vs. primary vaccine doses, we analyzed the levels of serum SARS-CoV-2 S protein-specific IgG4 Abs known to be associated with an immunoregulatory or tolerogenic immune state, particularly in the context of chronic antigen exposure [37,38,39]. Our results revealed a gradual yet consistent increase in the levels of SARS-CoV-2 S protein-specific IgG4 Abs over the course of this study that reached significance by the 15 m time point (Figure 9A). This observation is due to significantly higher levels of SARS-CoV-2 anti-S IgG4 Abs in the individuals who received the mRNA-1273 vaccine but not the BNT162b2 vaccine at the 15 m time point (Figure 9B). Thus, these findings identify one potential cause that might contribute to the waning or dampening of Ab responses to SARS-CoV-2 mRNA vaccines, though further investigation is needed to confirm this possibility.

## 4. Discussion

Our study has confirmed a robust immune response associated with SARS-CoV-2 mRNA vaccines, which was characterized by significant increases in SARS-CoV-2 S IgG (but not IgA and IgM) Ab levels following primary vaccine doses. This observation is consistent with previous research indicating that COVID-19 vaccines effectively induced high levels of IgG Abs against the SARS-CoV-2 S protein [40,41,42,43]. Additionally, we observed that SARS-CoV-2 IgG Ab levels declined six months post-vaccination and were moderately boosted by additional/booster vaccine doses, which is in agreement with other studies documenting the waning of vaccine-induced Abs over time [44,45,46,47].

Our observation of the low-level and short-lived but significant increases in SARS-CoV-2/CCCoV N protein-specific IgG Abs contrasts with some findings suggesting that mRNA vaccines generate only a SARS-CoV-2 S protein-specific response [47,48,49]. However, there is mounting evidence that the SARS-CoV-2 S protein carries numerous immunodominant antigenic epitopes capable of inducing Abs cross-reactive with other unrelated viruses (including influenza A virus), bacteria, and even various human/mammalian tissues [50,51,52,53,54,55], which is consistent with our current data. Specifically, antigenic similarity (~45%) was reported to exist between SARS-CoV-2 S glycoprotein and the human protein angiotensin-I [56]. Furthermore, a *Streptococcus salivarius* protein, RSSL-01370, was shown to contain regions with significant homology to the SARS-CoV-2 S receptor-binding domain, and mice immunized with it produced anti-Spike IgG Abs in their serum [57]. While most studies only focus on anti-SARS-CoV-2 S responses associated with COVID-19 vaccines, some that studied anti-S and anti-N report heterologous B and T cell activation following COVID-19 vaccination. For example, Assis and colleagues’ data demonstrated that there was an increase in SARS-CoV-2 N Ab prevalence after the initial vaccine roll-out in December of 2020, but this finding was not explained [58]. Fraley and colleagues also showed an increase in SARS-CoV-2 N antibodies in seropositive individuals vaccinated with COVID-19 mRNA vaccines [59]. This, and the fact that there were no subsequent increases in the N-specific antibodies, is highly consistent with what we see in our current study. Another clinical study detected cross-reactive T cell receptor repertoires in antigen-experienced T cells recognizing SARS-CoV-2, measles–mumps–rubella, or tetanus–diphtheria–pertussis epitopes [60]. So, while not very widely discussed/acknowledged, there is a modest increase in anti-SARS-CoV-2 N antibodies after the initial dose of COVID-19 mRNA vaccines. Some participants in our study cohort also could have had recent or ongoing undiagnosed CCCoV/SARS-CoV-2 infections that were mild or asymptomatic but that could prime their specific and cross-reactive immune responses. It is also conceivable that some individuals’ immune systems might generate a broader range of Ab responses in reaction to the vaccine by various mechanisms.

In our HCW cohort, SARS-CoV-2 S protein-specific IgG4 Ab levels increased significantly by the end of the study period, which is consistent with previous findings for COVID-19 mRNA vaccines [2,61,62]. While human IgG1 and IgG3 Abs are mostly associated with antiviral/protective immune responses, IgG4 Abs are known to be associated with poor Fc functional activity, decreased IgG1 and IgG3 levels, and a shift toward a tolerogenic immune response [2,61]. Consistent with our data, IgG4 class switch and decreased Fc functional capacity were observed following mRNA (mRNA-1273/BNT162b2) but not recombinant protein-based (Novavax) vaccination against SARS-CoV-2 in adults and children [39,62,63,64,65,66,67]. Additionally, this study further corroborates our data demonstrating higher IgG4 levels in those receiving the mRNA-1273 vs. BNT162b2 vaccine (Figure 9B). IgG4-biased immune response results from antigen persistence, increased antigen doses, or repeated exposure to viral components, and most evidence shows that it is not associated with improved viral clearance, in contrast to IgG1 and IgG3 [37,65]. So, it is consistent that the mRNA-1273 vaccine delivering 100 µg of mRNA/dose (vs. 30 µg/dose of the BNT162b2 vaccine) ultimately led to significantly higher IgG4 Ab levels. Thus, while the induction of IgG4 may offer benefits in terms of reducing inflammation and enhancing tolerance, it also raises questions about the potential interference with antigenic clearance and the long-term vaccine efficacy.

Further, the individuals with confirmed SARS-CoV-2 infections during the study period showed increased SARS-CoV-2 and cross-reactive Ab responses compared with those who had been vaccinated only. This is consistent with previous observations that natural infection, followed by vaccination, may provide a broader and more robust Ab response, potentially due to the broader epitope exposure during natural infection leading to enhanced affinity maturation and, possibly, to a more diverse and robust Ab repertoire [68,69]. Upon vaccination, these primed immune cells respond more vigorously, producing Abs that not only target the vaccine antigens but also other related CoVs [70]. Notably, our data also revealed that Vax inf individuals exhibited higher levels of cross-reactive Abs against CCCoVs, suggesting that natural infection primes immune memory in a way that broadens the response related to coronaviruses. This suggests that hybrid immunity not only strengthens SARS-CoV-2-specific responses but may also enhance cross-protection against endemic CoVs.

Finally, we did not observe a negative correlation between pre-existing CCCoV and vaccine-induced SARS-CoV-2 Abs, suggesting that the former do not interfere with the vaccine-induced immune response to SARS-CoV-2. This contrasts with the findings of at least one study [29], in which researchers investigated the efficacy of different immunization regimens in mice, comparing those receiving two doses of SARS-CoV-2 spike protein to those first immunized with a CCCoV S protein followed by SARS-CoV-2 S protein immunization. The findings demonstrated that the second group had decreased SARS-CoV-2 S-specific Ab levels, based on which the authors concluded that pre-existing CCCoV-induced Abs may inhibit SARS-CoV-2 Ab development following immunization. However, such a study design represents a significant limitation because it basically compared Ab responses associated with two doses vs. one dose of the SARS-CoV-2 S protein. Also, the second immunization took place 26 days after the initial one, resulting in IgA and IgG/virus neutralization titers being at peak, which is not representative of the variable but generally low levels of CCCoV Abs observed in our study cohort. In contrast, our finding is supported by several experimental and clinical studies [18,19,71]. Furthermore, we observed a positive correlation between the levels of the pre-existing CCCoV anti-N Ab response and certain SARS-CoV-2 Abs post-vaccination. This correlation was more pronounced for IgM and IgA SARS-CoV-2 Abs against the N protein, while it was minimal for IgG Abs. These findings are consistent with previous reports that IgM exhibits the highest level of cross-reactivity, followed by IgA and then IgG [29], and the fact that COVID-19 mRNA vaccines mostly induce SARS-CoV-2 S protein-specific IgG Abs. Surprisingly, although high SARS-CoV-2 IgG Ab levels are detected post-vaccination, we did not observe high cross-reactivity with CCCoVs, which may be due to differences in antigenic specificity and the immunodominance of the SARS-CoV-2 S protein.

Our study is the first and, to our knowledge, the only longitudinal study spanning 25 months to comprehensively measure three Ab isotypes (IgG, IgA, and IgM) against SARS-CoV-2 and common cold coronaviruses (CCCoVs) using both anti-S and anti-N peptide/protein targets, surpassing many previous studies [72,73,74,75] with shorter durations. Additionally, we used peptides from conserved N protein regions capable of recognizing cross-reactive antibodies against CCCoV belonging to the *Alphacoronavirus* and *Betacoronavirus* genera. These aspects contribute to the originality and significance of our study. The longitudinal design allowed us to profile the long-term immune response to SARS-CoV-2 mRNA vaccines in the context of pre-existing (post-infectious) antibodies against SARS-CoV-2 and CCCoVs. This provided a more comprehensive picture and in-depth understanding of SARS-CoV-2 immunity rather than focusing solely on the immunological response to SARS-CoV-2 S alone.

Regarding our cohort size, while the sample size may be smaller than some large-scale population studies, our extended follow-up period and detailed immune profiling provide high-resolution insights into Ab dynamics over time. This study serves as a hypothesis-generating investigation that can guide larger future studies with broader populations and aid in the development of optimized vaccines.

Our in-house ELISA was carefully designed and validated (using pathogen-specific antisera and clinical samples from several different patient cohorts, including pre-COVID-19, SARS-CoV-2-vaccinated, SARS-CoV-2-infected, and SARS-CoV-2-infected-and-vaccinated) and was shown to possess high specificity and reproducibility in measuring Ab responses [32,76]. Additionally, peptide-based ELISAs allow for the targeted evaluation of immune responses to specific viral epitopes, which is particularly relevant when studying responses to different coronavirus strains. Finally, our SARS-CoV-2 peptides provide variant of concern (VoC)-independent results, as the peptide sequences are 100% conserved with no mutations and deletions across different VoCs from various years based on multiple sequence alignment using MEGA10 with representative SARS-CoV-2 sequences from NCBI databases, ensuring that the results are robust and reliable across evolving strains of SARS-CoV-2.

This combination of extended observation, detailed immune profiling, and customized antigen selection enhances this study’s novelty and significance in understanding long-term immunity following SARS-CoV-2 vaccination. Unlike short-term experimental models, clinical studies like ours provide real-world insights that complement mechanistic research. While experimental models offer controlled conditions, they do not always capture the complexity of human immune responses over time and the impact of existing background cross-reactive Abs to CCCoVs.

## 5. Limitations of This Study

A limitation of this study is the absence of IAV vaccination/infection data for the cohort. The study design did not originally include IAV vaccine status or RT-PCR testing for IAV infections, making it impossible to confirm past exposures. Without this information, we were unable to rule out the potential impact of IAV vaccination on the participants’ Ab profiles. Also, we could not rule out the possibility of asymptomatic, undiagnosed, or unreported CCCoV or SARS-CoV-2 infections occurring alongside vaccination, particularly given the ongoing outbreak context. Individual immune responses can vary significantly due to factors such as comorbidities, genetics, and differences in immune history. This study did not account for all potential confounders, such as underlying health conditions, due to data limitations. This study focused on HCWs, a population with high exposure to SARS-CoV-2 and early access to vaccines. While this cohort (with a small sample size) provides valuable insights into long-term immunity, the findings may not be fully generalizable to the broader population, particularly individuals with different exposure risks, health conditions, or vaccination schedules. Additionally, considering the timing of sampling, most study participants had prior exposures to SARS-CoV-2, such that their immune responses to COVID-19 vaccines would differ from those of naïve individuals. Finally, while we had 12 confirmed SARS-CoV-2 infections in our cohort, we cannot rule out that there were additional (mild or asymptomatic) infections not accompanied by clinical diagnosis. Due to the observational nature of this study and the limited data available, we were unable to definitively establish a causal link between vaccination and the observed cross-reactivity, which warrants further investigation in future studies. Despite these limitations, this study provides a robust and extended evaluation of Ab responses to SARS-CoV-2 and CCCoVs over multiple years. Of significance, this study confirms that pre-existing CCCoV immunity does not negatively impact immune responses to mRNA SARS-CoV-2 vaccines in a short- or longer-term period.

## 6. Conclusions

In summary, this study demonstrates that the low levels of Abs against endemic CoVs do not significantly enhance or inhibit the development of an Ab response post-mRNA vaccination in healthy individuals. Further, our findings suggest that pre-existing IgM Ab against CCCoVs may serve as a biomarker for a more robust Ab response to SARS-CoV-2 mRNA vaccination. Importantly, a combination of SARS-CoV-2 infection and vaccination resulted in a more pronounced increase in CCCoV Ab levels compared to mRNA vaccination alone. This study also indicates that vaccine-induced Ab responses wane over time, potentially due to increasing IgG4 Ab levels. Our data also corroborate the prior evidence that COVID-19 mRNA vaccines can elicit variable levels of immunological cross-reactivity with unrelated antigens and viruses, but we cannot rule out asymptomatic infections by these viruses. Likewise, we cannot rule out that some of the participants received IAV vaccines during the study period. Finally, our results confirm that the COVID-19 mRNA vaccine-induced response is dominated by SARS-CoV-2 S-specific IgG Abs, while SARS-CoV-2 infection boosts the levels of SARS-CoV-2-specific and cross-reactive Abs against the S and N proteins. Lastly, our hypothesis-generating observations provide real-world insights into how Ab responses evolve, highlighting areas for further mechanistic investigation.

## Figures and Tables

**Table 1:** Time point, vaccination, and SARS-CoV-2 infection summary.

Time Point	Sample Collection Period	Number of Individuals (n)	Details
Pre-vaccination (Pre)	April–May 2020	64	Collected approximately 7–8 months prior to vaccination.
Post-vaccination (Vax1)	13–33 days after 1st dose (Median = 21)	61	Collected after the first vaccine dose. Interquartile range (IQR) = 19–23 days.
Post-vaccination (Vax2)	20–31 days after 2nd dose (Median = 26)	62	Collected after the second vaccine dose. IQR = 22–28 days.
6-month post-vaccination (6 m)	6 months post-vaccination	57	Collected at the 6-month follow-up.
9-month post-vaccination (9 m)	9 months post-vaccination	55	Collected at the 9-month follow-up.
12-month post-vaccination (12 m)	12 months post-vaccination	54	Collected at the 12-month follow-up.
15-month post-vaccination (15 m)	15 months post-vaccination	49	Collected at the 15-month follow-up.
Booster dose (BNT162b2/mRNA-1273)	1–80 days prior to the 9 m time point	23	Total of 23 individuals; BNT162b2 (n = 17), mRNA-1273 (n = 6). Median = 18 days, IQR = 7–29 days before 9 m time point.
Second booster (BNT162b2)	Timeframe unknown	2	Collected after the second booster dose, timeframe not available.
Vaccine type information	-	-	Vax1: 61 individuals (mRNA-1273 n = 28, BNT162b2 n = 33), Vax2: 62 individuals (mRNA-1273 n = 28, BNT162b2 n = 34), boosters: 23 individuals (BNT162b2 n = 17, mRNA-1273 n = 6), 2 received second booster doses (BNT162b2).
SARS-CoV-2 infections	-	12	Ten confirmed infections 1–9 months prior to 1st dose, one infection within 12 days post-Vax1 (but prior to Vax 1 sampling), one breakthrough infection ~6 months post-Vax2.

## Data Availability

The data that support the findings of this study are available from the corresponding author, A.N.V., upon reasonable request.

## Supplementary Materials

The following supporting information can be downloaded at https://www.mdpi.com/article/10.3390/vaccines13050547/s1. Figure S1: (**A**) SARS-CoV-2-specific IgG Ab titers in individuals that received primary vaccine series only or primary vaccine series plus 1 or more booster doses. (**B**) Comparison of SARS-CoV-2 anti-S IgG antibody titers: individuals with prior infection and booster vaccination (Infected+ Boosted), individuals with prior infection but no booster vaccination (Infected + Non-Boosted), individuals with no prior infection and booster vaccination (Non-infected+ Boosted), and individuals with no prior infection and no booster vaccination (Non infected+ Non-Boosted). Figure S2: SARS-CoV-2-specific IgG Ab titers in individuals that received different mRNA vaccines (mRNA-1273 vs. BNT162b2).
