# Supplementary material for: Circulating Antibodies Against Common Cold Coronaviruses Do Not Interfere with Immune Responses to Primary or Booster SARS-CoV-2 mRNA Vaccines
Source: Vaccines (Basel). 2025 May 21;13(5):547. doi: 10.3390/vaccines13050547 (PMC12115401; doi:10.3390/vaccines13050547)
Supplement: Supplementary file 1 [file vaccines-13-00547-s001.zip › vaccines-3589975-supplementary.pdf]

Supplemental data

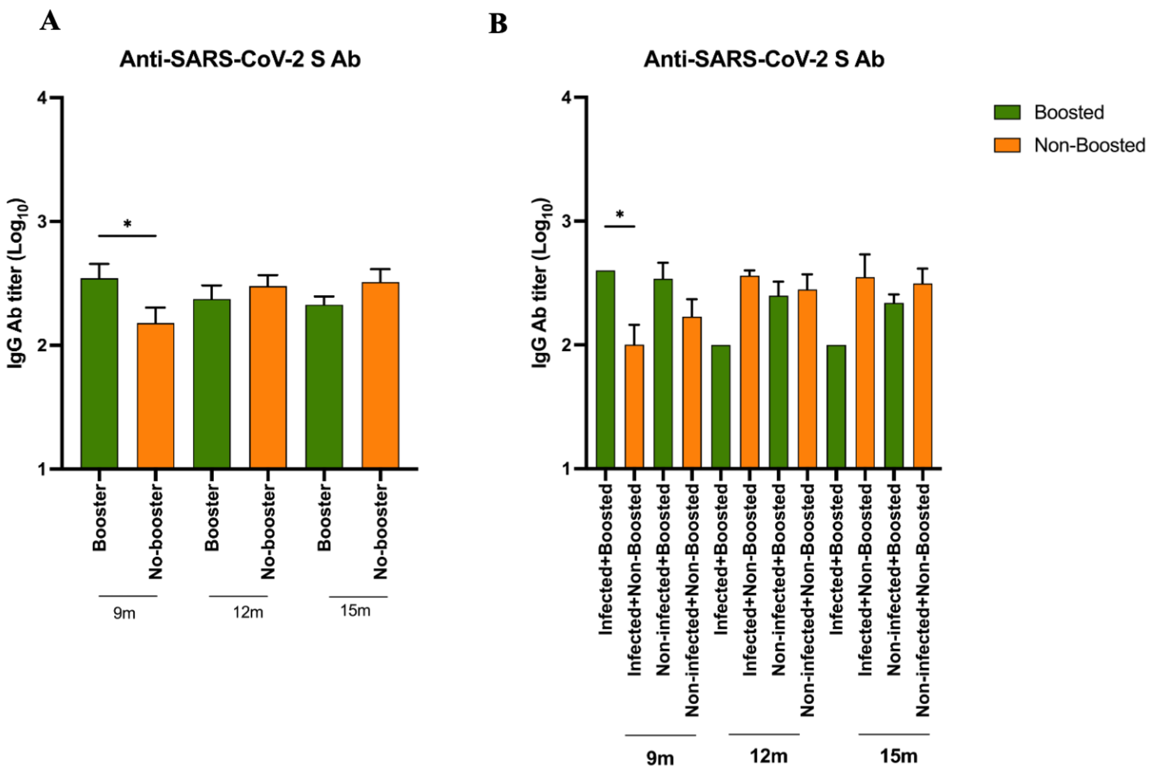

Figure S1. (**A**) SARS-CoV-2-specific IgG Ab titers in individuals that received primary vaccine series only or primary vaccine series plus 1 or more booster doses. (**B**) Comparison of SARS-CoV-2 anti-S IgG antibody titers: individuals with prior infection and booster vaccination (Infected+ Boosted), individuals with prior infection but no booster vaccination (Infected + Non-Boosted), individuals with no prior infection and booster vaccination (Non-infected+ Boosted), and individuals with no prior infection and no booster vaccination (Non infected+ Non-Boosted). Significant differences indicated by asterisks (*$p < 0.05$). The data illustrate the impact of both booster vaccination and prior infection on antibody responses.

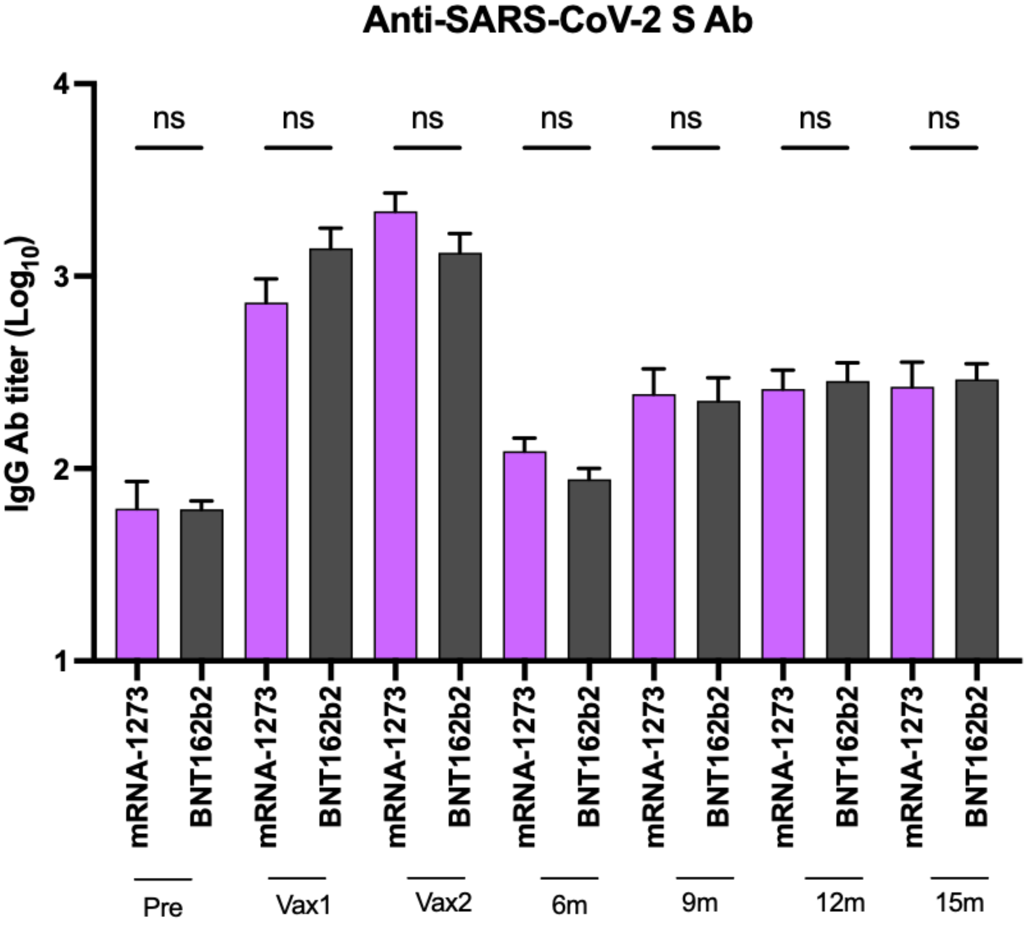

Figure S2. SARS-CoV-2-specific IgG Ab titers in individuals that received different mRNA vaccines (mRNA-1273 vs. BNT162b2). NS denotes that the results are not statistically significant.
